# Differential Modulation of Transcription Factors and Cytoskeletal Proteins in Prostate Carcinoma Cells by a Bacterial Lactone

**DOI:** 10.1155/2018/6430504

**Published:** 2018-05-09

**Authors:** Senthil R. Kumar, Jeffrey N. Bryan, Amanda M. Eaton, Katherine L. Robinson, Saivaroon Gajagowni

**Affiliations:** ^1^Comparative Oncology, Radiobiology and Epigenetics Laboratory, College of Veterinary Medicine, Columbia, MO 65211, USA; ^2^Department of Surgery, Ellis Fischel Cancer Centre, School of Medicine, University of Missouri, Columbia, MO 65201, USA

## Abstract

The present study tested the effect of a bacterial lactone N-(3-oxododecanoyl)-homoserine lactone (C12-HSL) on the cytoskeletal and transcriptional genes and proteins in prostate adenocarcinoma (PA) cells (DU145 and LNCaP) and prostate small cell neuroendocrine carcinoma (SCNC) PC3 cells including their cellular viability and apoptosis. Our data indicate that cell migration and colony formation were affected in the presence of C12-HSL. C12-HSL induced apoptosis and altered viability of both PA and SCNC cells in a concentration dependent manner as measured by fluorescence and chemiluminescence assays. Compared to PCa cells, noncancerous prostate epithelial cells (RWPE1) were resistant to modification by C12-HSL. Further, the viability of PC3 cells in 3D matrix was suppressed by C12-HSL treatment as detected using calcein AM fluorescence in situ. C12-HSL treatment induced cytoskeletal associated protein expression of vinculin and RhoC, which may have implications in cancer cell motility, adhesion, and metastasis. IQGAP protein expression was reduced in DU145 and RWPE1 cells in the presence of C12-HSL. C12-HSL decreased STAT3 phosphorylation in DU145 cells but increased STAT1 protein phosphorylation in PC3 and LNCaP cells. Overall, these studies indicate that C12-HSL can trigger changes in transcription factors and cytoskeletal proteins and thereby modulate growth and migration properties of PCa cells.

## 1. Introduction

Bacterial quorum sensing (QS) is a process through which bacterial cells communicate to coordinate their response as a function of population density [1]. For instance, gram negative bacteria such as* Pseudomonas aeruginosa* employ N-(3-oxododecanoyl)-homoserine lactone (C12-HSL) as a signaling molecule for QS [2]. Studies of HSLs indicate that these molecules not only function as a communication sensor in different bacteria but can also influence eukaryotic cell behavior [2]. However, the mechanism of action and subsequent effect of HSL compounds on eukaryotic targets are still emerging. It has been suggested that different HSLs act through multiple signaling pathways and interact with cell membrane components [3, 4]. For instance, C12-HSL having a long acyl chain and intact homoserine lactone ring structure can associate with cell membrane phospholipids in Jurkat T-cells [4]. Another study reported that, upon entering the mammalian cells [5], C12-HSL may utilize intracellular nuclear peroxisome proliferator activated receptors (PPARG) to affect transcriptional activity and NF-*ƙ*B signaling [6]. The tight junctions present in epithelial cells can be altered by C12-HSL possibly through activating signaling pathways such as MAPK which include extracellular regulated protein kinases (ERK) and p38 [7, 8].

Transcriptional factors such as signal transducer and activators (STAT) STAT3 and STAT1 can function by activating different but overlapping set of genes [9, 10]. While STAT3 is an oncogene [11], STAT1 may function as a tumor suppressor [12]. In prostate carcinoma, STAT3 may play a critical role in maintaining the cancer stem cells (CSC) [13]. STAT3 also can directly inhibit p53, and, in tumors with constitutive STAT3 activation, it may activate HIF-1*α* and VEGF [13]. Given the wide involvement in various pathways, STAT3 can be an important signaling component in prostate carcinoma (PCa); efforts aimed at targeting this protein for cancer treatment are steadily increasing. On the other hand, STAT1 also plays a critical role by inducing antiproliferative and proapoptotic activities which hampers tumor growth [14]. A previous report suggests that STAT3 could be downmodulated by C12-HSL in breast carcinoma [15]. However, C12-HSL actions on STAT3 or STAT1 in PCa cells are not known. Moreover, given the reciprocal regulation and opposing functions between STAT1 and STAT3 [16], how interfering with one protein will affect the other in the presence of C12-HSL remains unknown.

The dysregulation of cytoskeletal protein network plays a critical role in the progression of solid tumors including PCa [17]. Increased motility and invasiveness of tumor cells are possible due to reduced cell adhesion. The structural adhesion proteins, such as integrins, connect to actin by docking proteins including vinculin, paxillin, and talin with GTP binding signaling proteins that modulate organization of the actin cytoskeleton [17]. For instance, vinculin present in focal adhesions and cell-adherence junctions provides a mechanical link and affects the turnover of contractility and adhesion proteins [18]. Vinculin supports anchorage-dependent cell growth decreasing cell motility and has been suggested to function as a tumor suppressor [19]. Another GTPase protein RhoC promotes polarized cell migration and invasion by controlling cell spreading and Rac1 activation around the cell periphery, hence restricting lamellipodial broadening [20]. It has also been reported that RhoC in association with IQGAP, a scaffold protein, stimulates the migration of gastric cancer cells [21]. C12-HSL was shown to alter IQGAP protein in epithelial cells [22] and thereby decrease cell migration. However, the expression of various cytoskeletal proteins among different epithelial or SCNC prostate tumor epithelial cells and the effect of C12-HSL on these proteins in PCa cells remain unexplored.

In the present study, we report the effect of C12-HSL on the viability and apoptosis of human PCa cells, along with its effects on cellular migration and colony forming ability. C12-HSL affected the cellular properties in a concentration dependent manner in different PCa cell types. C12-HSL reduced the viability of PCa cells grown in 3D matrix. We also found differences in the expression of different cytoskeletal proteins in PCa cells with variable susceptibility to C12-HSL. Further, C12-HSL modulates the transcription factor proteins STAT3, STAT1, and cyclin dependent kinase inhibitor 1A (CDKN1A, P21^waf1/cip1^) including their phosphorylation status, depending on the PCa cell type.

## 2. Materials and Methods

### 2.1. Materials

Human prostate adenocarcinoma cells (DU145 (Androgen receptor (AR −ve) and LNCaP (AR +ve) and small cell neuroendocrine carcinoma cells (SCNC) (PC3 (AR −ve)) [23] and normal prostate epithelial cells (RWPE1) were purchased from American Type Culture Collection (ATCC), Manassas, VA. C12-HSL was purchased from Cayman chemicals (Ann Arbor, MI). Antibodies for different proteins were purchased from Cell Signaling Technologies (Danvers, MA). Phospho-CDKN1A (pCDKN1A) antibody (Thr-145) was procured from Santa Cruz Biotechnology (Dallas, TX). Cellular viability, cytotoxicity, and apoptosis Triplex assay kit were purchased from Promega Corporation (Madison, WI). Calcein AM was purchased from R&D systems (Minneapolis, MN). Gene primers for qRT-PCR assays were purchased from Integrated DNA Technologies (Coralville, IA). All other materials used were purchased from Fischer Scientific unless mentioned otherwise.

### 2.2. Cell Culture

PCa cells were grown in complete RPMI medium with 10% fetal bovine serum (FBS) and gentamicin at 37°C with 5% CO2. RWPE1 cells were grown in keratinocyte free media with EGF and bovine pituitary extract.

### 2.3. Cell Viability, Cytotoxicity, and Apoptosis

Cell viability, cytotoxicity, and apoptosis of PCa cells in the presence or absence of C12-HSL were performed using a Triplex assay. The stock solution of C12-HSL was made in 100% DMSO and further diluted in 1x PBS or the culture media for subsequent addition to the cells. The DMSO concentration in the assays was <0.01%. The PCa cells were grown in 96 well plates at a density of 5 × 10^3^ cells/well and treated with C12-HSL (0–200 *μ*mol/L) or DMSO vehicle (<0.01%). The cells were further incubated for 24 h. After incubation, Triplex assay reagent (Promega, Madison, WI) was added to the cell plates and further incubated for the recommended time according to the manufacturer's instructions. Cell viability and cytotoxicity were measured by fluorescence at 400_Ex_/505_Em_ and 485_Ex_/520_Em_ wavelengths, respectively, using a Tecan Safire fluorescence and absorbance plate reader (Mannedorf, Switzerland). For apoptosis through caspase activation, the plates were evaluated using a Veritas™ microplate luminometer (Turner Biosystems, Sunnyvale, CA). Each experiment was performed in triplicate.

### 2.4. Colony Forming Assay

A colony forming assay was utilized to test PCa cell survival as described previously [24]. C12-HSL (50 *μ*mol/L) was added to PCa cells grown in culture plates and incubated for 24 h. In parallel, cells without the addition of C12-HSL treatment were also grown. Subsequently, the cells were washed with 1x PBS, lifted from the culture plates using TrypLE, and tested with Trypan Blue dye for viable cells. Initially, we tested the ability of these cells to form efficient colonies and based on that we plated the cells accordingly for RWPE1 and PCa cells. C12-HSL treated and untreated cells were plated in 35 mm cell culture dishes at a density of 2.5 × 10^2^ cells (DU145), 5 × 10^2^ cells (PC3), 5 × 10^2^ cells (LNCaP), and 7 × 10^3^ cells (RWPE1) per plate. The live cell count numbers were maintained similar in treated and untreated cells. The cells were grown for ten days at 37°C. The cells were fixed with 70% methanol and stained with 0.5% crystal violet. The colonies were counted as described previously [24]. Colonies, defined as groups of ≥20 cells, were identified and designated as a single colony. The plating efficiency was calculated based on number of cells to colony numbers and was about 75%.

Wound healing assays [25] were done to assess the cell migration ability. PC3 and DU145 cells (2.5 × 10^4^ cells/well) were seeded in six well plates and grown until a cell monolayer was formed. To inhibit cell proliferation, they were treated with mitomycin-C for 2 h. Followed by this, a similar sized scratch was made with a sterile pipette tip across the center of each well and imaged at baseline and after 48 h (PC3, DU145, and RWPE1), before and after C12-HSL (50 *μ*mol/L) treatment. The image was acquired using Olympus CK40 phase contrast inverted microscope (Center Valley, PA), and the image gap area was measured using ImageJ (NIH, USA) software. An arbitrary number of 1 was assigned to the wound area at 0 h and the 48 h values were relative to the baseline value. The assay was repeated twice on separate days with different cell passages.

### 2.5. Cell Viability in 3D Cultures

For growing PCa cells on 3D matrix, prechilled chamber slides were coated with a thin layer of Growth Factor reduced Matrigel matrix (BD Biosciences, Sane Jose, CA) maintained at 4°C. The cells were suspended in serum-free DMEM/F12 medium and added to matrigel (4 : 1 matrix to media) maintained on ice. From this mixture, a total of 50 *μ*l (10^4^ cells/well) was transferred to the matrigel precoated chamber slide wells. The cells were grown for 7 days and the cell growth medium was replenished once every two days. After 7 days, C12-HSL (75 *μ*mol/L) was added consecutively for 5 days with change in cell media and maintained at 37°C for the duration of the study. After the treatment, the cells were washed with 1x PBS and were imaged in an Olympus CK40 phase contrast inverted microscope to image the morphology of the cells. In separate experiments, Calcein AM dye (Ex/Em = 494/520 nm) (Biotium, Hayward, CA) was added to the cells after C12-HSL treatment and incubated for 2 h at 37°C to test the cell viability and cell membrane integrity. The cells were imaged using an Olympus CK40 fluorescent microscope. The corrected total cell fluorescence (CTCF) was calculated using ImageJ (NIH) using the following formula: CTCF = Integrated Density − (Area of selected cell × mean fluorescence of background readings) [26].

### 2.6. Fluorescence Microscopic Analysis

Fluorescence microscopic analysis was performed to assess the actin filaments in PC3 cells without and with C12-HSL treatment using Acti-stain™ 488 fluorescent phalloidin (Cytoskeleton Inc., Denver, CO). Cells were cultured in chamber slides with DMSO vehicle (<0.01%) or with C12-HSL (50 *μ*mol/L) in DMSO for 1 h, at 37°C and 5% CO2. Subsequently, the cells were then fixed with cold paraformaldehyde followed by addition of Triton-X 100 (0.1% in 1x PBS) to enhance membrane permeability. Cells were washed with phosphate buffered saline (PBS), labeled with phalloidin, and incubated for 1 h at RT. After washing with 1x PBS, cell nuclei were counterstained using 4′,6′-diamino-2-phenylindole (DAPI). The slides were visualized using a Nikon Eclipse E600 fluorescence microscope (Nikon Instruments Inc., Melville, NY).

### 2.7. Real-Time PCR

Total RNA from PCa cells treated with or without C12-HSL (75 *μ*mol/L for 6 h) were isolated using RNeasy kit (Qiagen, Valencia, CA). Complementary DNA (cDNA) was generated using iScript Reverse Transcript Supermix (Biorad, Hercules, CA) and subsequently for downstream qRT-PCR analysis. qRT-PCR was performed with CFX connect (Biorad) using iTaq Universal SYBR Green super mix. Relative quantification of mRNA expression was calculated using ΔΔCq method. Cq values above 32 were considered negative in these experiments. One-Way Analysis of Variance (ANOVA) was used to calculate the statistical significance. *P* value ≤ 0.05 was considered significant. The following gene primers were used for qRT-PCR.* STAT3* (F-CTGGGCTTTGGTGTTGAAATAG, R-CAGATCAAGTCCAGGGAGAAAG),* STAT1* (F-CCAAAGTATCAGGACGAGAATGA, R-CTACGTCAAGCAGTTCCCTAAA),* CDKN1A,* (F-TTAGCAGCGGAACAAGGAGTCAGA, R-ACACTAAGCACTTCAGTGCCTCCA),* GAPDH (F-GTCATCATCTCTGCTCCTTCTG, R-AAGAAGGTAGTGAAGCAGGC), Vinculin *(F-AGCCAGAACCAGATGAGTAAAG, R-GCCTGAGTGTAAAGGACCAA),* RhoC *(*F-*GGTCACACACCAGCACTTTA, R-TTGGAGCCTGTAGCCTTTATTC),* IQGAP1* (F-CCACATCCAAGACAGGCAATA, R-GGCATCCTCTGTGCTACTAAAG), and* cofilin1 *(*F-*GAGGTGAAGCGCAAGAA, R-GGTTGCATCATAGAGGGCATAG).

### 2.8. Western Blot for Protein Expression

Proteins were extracted from WT and PCa cells exposed to C12-HSL (75 *μ*mol/L for 24 h) using M-PER mammalian protein extraction reagent (Thermofisher, Rockford, IL). Protein concentrations were measured using the Coomassie Blue reagent (Thermofisher). For western blot analysis, equal amount of proteins (~40 *μ*g) was loaded on a 4–12% SDS-polyacrylamide gel to separate proteins. Subsequently, the proteins were transferred onto nitrocellulose membrane (Biorad) using a blotting apparatus (Invitrogen, Thermofisher) and a voltage of 30 mA at room temperature. After the transfer, the membrane was blocked with 5% BSA for 1 h. The membrane was further incubated with primary antibodies for Vinculin, RhoC, IQGAP1, cofilin1, STAT3, pSTAT3, STAT1, CDKN1A, pCDKN1A, and GAPDH, respectively, overnight at 4°C. After washing the membrane (×3) with Tris Tween buffered saline (1xTTBS), the membrane was incubated with a horseradish peroxidase (HRP) conjugated secondary antibody. After incubation, the membrane was washed (×3) with TTBS and the blot was imaged in a Kodak imaging station (Carestream Health, Rochester, NY). The protein band intensities were measured using ImageJ, image processing program (NIH), and the difference in the intensities was calculated by dividing the percent values of C12-HSL treated by untreated WT cell protein bands. The results are depicted as fold difference.

### 2.9. Statistical Analysis

The statistical analysis was performed by calculating means, standard deviation, and standard errors, where indicated. Student's *t*-test was used to calculate differences between groups. *P* value ≤ 0.05 was considered statistically significant. Statistical analysis and graphs were generated using GraphPad Prism 7 (GraphPad Software Inc., La Jolla, CA).

## 3. Results

### 3.1. C12-HSL Affected the Cellular Properties of PCa Cells

**Figure 1:**
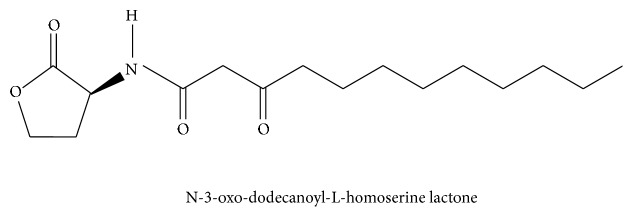
*Chemical structure of C12-HSL* (N-(3-oxododecanoyl)-homoserine lactone).

C12-HSL (Figure 1) has been reported to alter the cellular properties such as viability and apoptosis in different tumor cells [15, 24, 27]. However, the response to C12-HSL could vary depending on different tumor cell types. Therefore, it is intriguing to understand the mechanism of action of this compound on normal prostate epithelial and PCa cells. To determine the effect of C12-HSL on different PCa cellular properties, assays for viability, cytotoxicity, and apoptosis were performed with different concentrations of this compound and compared with untreated cells. A summary of the EC50 values for C12-HSL on different cellular properties is depicted in Table 1. The graphical representation of the assays is given in (Figure S1). Cellular viability decreased as a measure of C12-HSL concentration and had a half-maximal inhibitory concentration (EC_50_) varying in the range of 75–>100 *μ*mol/L among different cells. Similarly, the EC_50_ for cytotoxicity were in the range of 60–130 *μ*mol/L and that of apoptosis 75–100 *μ*mol/L. For further downstream studies, we utilized C12-HSL concentration around 50 or 75 *μ*mol/L unless indicated otherwise, in order to avoid any nonspecific effects of this compound at higher concentrations.

### 3.2. C12-HSL Inhibited the Clonogenic Ability of PCa Cells

**Figure 2:**
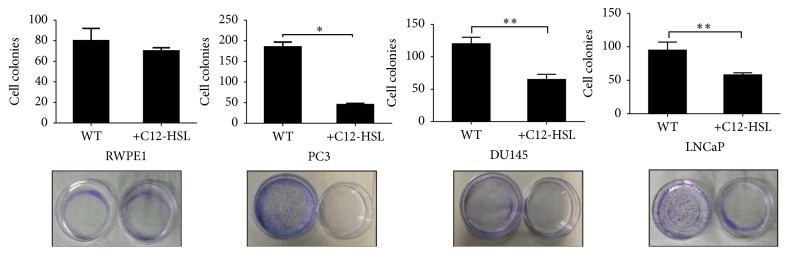
*Colony formation assay*. C12-HSL treated (50 *μ*mol/L) and untreated cells were plated in 35 mm cell culture dishes at a density of 2.5 × 10^2^ cells (DU145), 5 × 10^2^ cells (PC3), 5 × 10^2^ cells (LNCaP), and 7 × 10^3^ cells (RWPE1) per plate. The live cell count numbers were maintained similar in treated and untreated cells. Colonies, defined as groups of ≥20 cells, were identified and designated as a single colony. The plating efficiency was calculated based on number of cells to colony numbers and was about 75%. ^*∗*^*P* < 0.01; ^*∗∗*^*P* < 0.025.

Given the effect of C12-HSL on the viability of different PCa cells, we next investigated the clonogenic survival of the cells in a colony forming assay. Clonogenic ability of PC3, DU145, LNCaP, and RWPE-1 cells was tested in the presence of C12-HSL (50 *μ*mol/L) (Figure 2). The concentration of C12-HSL was maintained at 50 *μ*M which is lower than the observed IC_50_ values (~75–85 *μ*mol/L) to maintain nontoxic dose level to the cells. The colony formation of all the PCa cells was reduced in the presence of C12-HSL by 76% (PC3, *P* = 0.009), 46% (DU145, *P* = 0.01), and 40% (LNCaP, *P* = 0.015), respectively. The colony formation of RWPE1 cells was less affected (5%, *P* > 0.05) compared to PCa cells.

### 3.3. PCa Cell Growth in 3D Matrix

**Figure 3:**
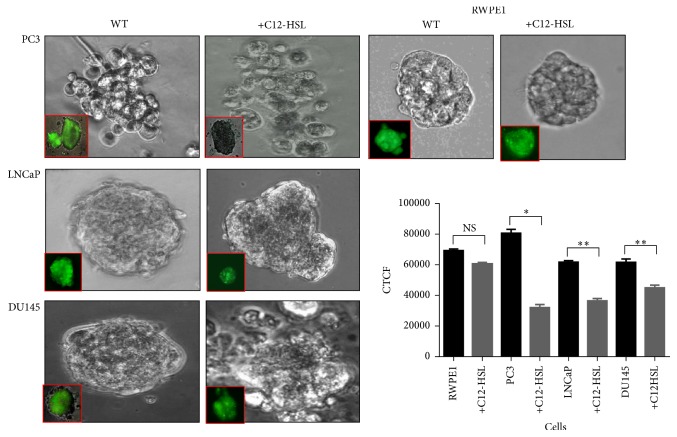
*3D Cell viability assay*. Different cells (10^4^ cells/well) were grown in matrigel precoated chamber slide wells. The cells were grown for 7 days with change in cell medium once every two days. After one week, C12-HSL (75 *μ*mol/L) was added consecutively for 5 days with change in cell media and maintained at 37°C for the duration of the study. After C12-HSL treatment, the cells were washed with 1x PBS and were imaged in an Olympus CK40 phase contrast inverted microscope to image the morphology of the cells.* Inset*. As a separate experiment, Calcein AM dye (Ex/Em = 494/520 nm) (Biotium, Hayward, CA) was added to the cells after C12-HSL treatment and incubated for 2 h at 37°C to test the cell viability and cell membrane integrity. The cells were imaged using an Olympus CK40 fluorescent microscope. The fluorescence intensity measurements were created with ImageJ (NIH). The fluorescent intensities are depicted as CTCF values in the graph. ns, not significant; ^*∗*^*P* < 0.001 and ^*∗∗*^*P* < 0.025.

While 2D cell culture assays facilitate studying the cell behavior, using a 3D environment for examining the cellular behavior could be more relevant to study the effect of C12-HSL as it better approximates natural interaction with a basement membrane matrix (BMM). Further, the cells may behave differently in response to drug-like compounds in 2D culture versus 3D culture and the later growth conditions mimic the* in vivo* tumor growth. Therefore, we investigated the effect of C12-HSL on the viability of the cells in a three-dimensional matrix in the presence of C12-HSL. The cells grew as mostly round shaped tumor spheroids in the matrix. Upon treatment with C12-HSL, changes in the cell shape, integrity, and morphology were observed (Figure 3). In separate experiments, to further probe the effect of C12-HSL on the cellular integrity and viability and visualize such changes in situ, the cells were grown in BMM until the development of well-defined tumor spheroids. C12-HSL was added as described in the methods. After the treatment, Calcein AM, a cell permeant dye, was added and after incubation for 2 h and subsequent washing to remove excess dye, the cells were imaged in a fluorescence microscope (Figure 3, Inset). While the wild type (WT) cells retained fluorescence, the cells treated with C12-HSL had variable retention of the dye suggesting possible changes in their cellular integrity. However, no extensive change was noted in RWPE1 cell fluorescence indicating relatively less damage response to C12-HSL. The CTCF for the WT and C12-HSL are depicted in Figure 3.

### 3.4. Cell Migration

**Figure 4:**
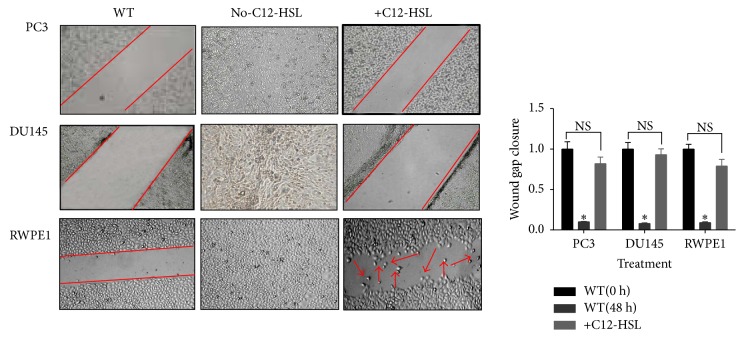
*C12-HSL inhibits cell migration*. Wound healing assays with PC3, DU145, and RWPE1 cells in the presence of C12-HSL (50 *μ*mol/L) for 48 h. In untreated cells, the wound closure was complete. In the presence of C12-HSL no wound gap closure was observed. ns, not significant; ^*∗*^*P* < 0.001. Red arrows depict the movement of RWPE1 cells into the wound space.

Due to the modulation of cytoskeletal protein expression by C12-HSL, we next investigated the cellular migration of PCa and RWPE-1 cells. PC3 and DU145 along with normal RWPE1 cells were used to observe the cellular migration in the absence and the presence of C12-HSL. While the migration of cells and wound closure were complete in the absence of C12-HSL in PCa cells, the cell migratory behavior diminished in the presence of this compound (Figure 4). A similar pattern was also observed in LNCaP cells (data not shown). The C12-HSL also affected the migration of RWPE1 cells; however, we noted the migration of few RWPE1 cells into the wound, unlike the PCa cells.

### 3.5. qRT-PCR Analysis

**Figure 5:**
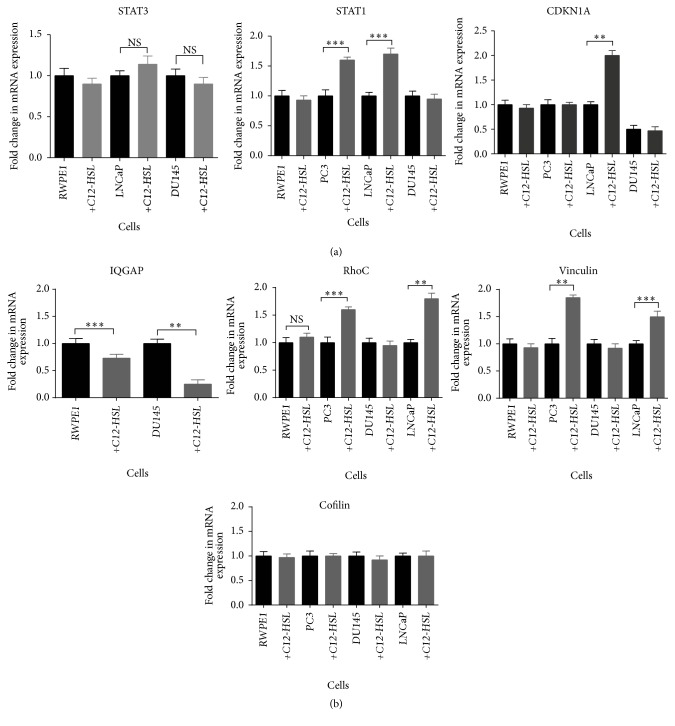
*Effect of C12-HSL on various mRNA expression*. (a) Changes in mRNA levels of* STAT3, STAT1,* and* CDKN1A,* and (b)* IQGAP, RhoC, vinculin, and cofilin* were analyzed by qRT-PCR with and without C12-HSL (75 *μ*mol/L). ns, not significant; ^*∗∗*^*P* < 0.025 and ^*∗∗∗*^*P* < 0.05. 18sRNA was used as an internal control.

Due to the multifaceted roles of STAT3 and opposing roles between this protein and STAT1 [13, 28] along with previous reports that C12-HSL can downmodulate STAT3 in breast carcinoma cells [15], we investigated the effect of C12-HSL on changes in both gene and protein expression of these molecules in PCa cells. The changes in mRNA expression of* STAT3*,* STAT1,* and* CDKN1A* upon addition of C12-HSL were analyzed by qRT-PCR in the normal and PCa cells (Figure 5(a)).* STAT3* mRNA expression was very low or undetectable in PC3 cells. Both LNCaP and DU145 wild type (WT) cells including RWPE1 expressed* STAT3*. While a marginal increase (<1-fold, *P* > 0.05) in LNCaP* STAT3* expression upon addition of C12-HSL was observed, a decrease (<1-fold) was noted in DU145 cells and RWPE1 cells which was not significant (*P* = 0.05).* STAT1* mRNA expression was observed in WT RWPE1 and PCa cells. The expression increased more than onefold in PC3 (*P* = 0.042) and LNCaP (*P* = 0.038) cells in the presence of C12-HSL. However, no changes were noted in DU145 or RWPE1 cells.* CDKN1A* gene expression was observed in RWPE1, PC3, and LNCaP cells but very low in DU145 cells. Upon C12-HSL addition, a twofold increase in the expression was observed in LNCaP cells (*P* = 0.023), but other cells did not exhibit any changes in this mRNA.

Changes or dysregulation in cytoskeletal proteins may play role in cancer cell migration and adhesion properties. Cytoskeleton associated genes including* vinculin, RhoC, and cofilin1* were found to be expressed in all the WT PCa cells and RWPE1 cells.* IQGAP1*, another molecule which interacts with cytoskeletal components, and adhesion molecules were found to be expressed in RWPE1 and DU145 cells (Figure 5(b)) but had very low or no expression in PC3 and LNCaP cells (data not shown). Upon addition of C12-HSL*, IQGAP1* gene expression decreased in both RWPE1 (1.3-fold, *P* = 0.037) and DU145 cells (4 fold, *P* = 0.02).* RhoC* mRNA expression increased up to 0.5–1.5-fold in RWPE1 (*P* = 0.042), PC3 (*P* = 0.036) and LNCaP (*P* = 0.021). Vinculin mRNA levels were induced up to 0.5–1-fold in LNCaP (*P* = 0.041) and PC3 (*P* = 0.02) cells upon treating with C12-HSL. While* cofilin1* expression was observed in all the WT cells, no change in* cofilin1* expression was observed in C12-HSL treated cells.

### 3.6. Protein Expression Studies

**Figure 6:**
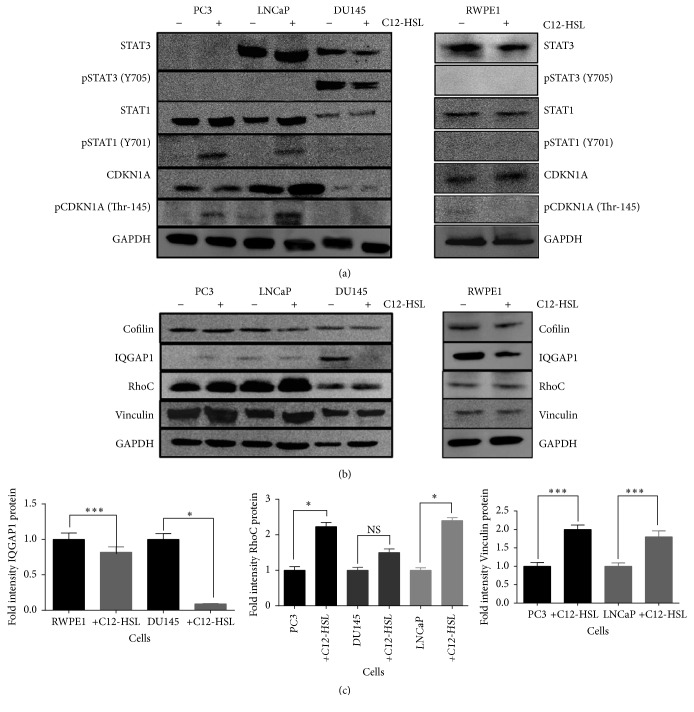
*C12-HSL modulation of different proteins*. (a) Western blot analysis for STAT3 (~80 kDa), pSTAT3 (Tyr 705; ~80 kDa), STAT1 (~81 kDa), pSTAT1 (Tyr 701) (~81 kDa), CDKN1A (~19 kDa), and pCDKN1A (Thr 145; ~19 kDa) in RWPE-1, PC3, LNCaP, and DU145 cells with or without treatment with C12-HSL (75 *μ*mol/L) for 24 h. (b) Western blot analysis for Cofilin, IQGAP1 (~195 kDa), RhoC (~23 kDa), and vinculin (~135 kDa) in RWPE-1, PC3, LNCaP, and DU145 cells with or without treatment with C12-HSL (75 *μ*mol/L) for 24 h. GAPDH (~38 kDa) was used as a control protein. (c) Graphs depicting the fold changes in different proteins. The intensity of the protein bands was measured using ImageJ program. Based on the raw data, the WT cells were assigned an arbitrary value of 1. Protein fold changes in C12-HSL treated cells were relative to WT cells. ^*∗*^*P* ≤ 0.001 and ^*∗∗∗*^*P* ≤ 0.05.

The difference in the protein expression of transcription factors STAT3 and STAT1, including CDKN1A, was analyzed by western blot in WT cells and in cells exposed to C12-HSL as described in methods. GAPDH was used as loading control (Figure 6(a)). While STAT3 was absent in PC3 cells, both LNCaP and DU145 cells as well as RWPE1 expressed this protein. A marginal increase (1 fold) in total STAT3 was observed in LNCaP while a threefold decrease was observed in DU145 cells in the presence of C12-HSL. No difference in total STAT3 status was observed in RWPE1 cells. Constitutive phosphorylation of STAT3 (pSTAT3) was noted only in WT DU145 cells which decreased (1.6-fold) upon C12-HSL addition. RWPE1 or LNCaP cells had neither constitutive or C12-induced pSTAT3. On the other hand, addition of C12-HSL seems to increase total STAT1 in both PC3 (1.3-fold) and LNCaP cells (1.86-fold) but not in DU145 cells. Total STAT1 appears to be low in DU145 relative to other two PCa cells. pSTAT1 was noted in PC3 and LNCaP cells upon C12-HSL addition but not in DU145 or RWPE1 cells.

CDKN1A protein was found to be expressed in PC3, LNCaP, and RWPE1 but very low in DU145 cells. In the presence of C12-HSL, we observed changes in the total protein expression in LNCaP (1.7-fold increase) cells but none in PC3, DU145, or RWPE1 cells. While constitutive phosphorylation of CDKN1A in PC3, LNCaP, and RWPE1 cells was low or absent, phosphorylation (Thr-145) was observed in all these cells. No CDKN1A phosphorylation in DU145 cells was evident (Figure 6(a)).

**Figure 7:**
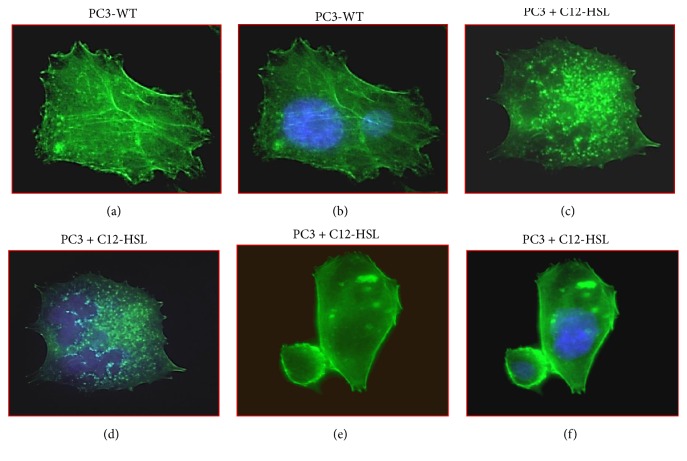
*Fluorescence microscopy analysis of actin in PC3 cells*. WT and C12-HSL treated (50 *μ*mol/L, 1 h at 37°C) PC3 cells were analyzed using Acti-stain 488 fluorescent phalloidin. WT PC3 (a and b) cells exhibited more organized actin filaments. However, C12-HSL treatment induced changes in the actin filaments and appeared more punctuated (c–f). Cell nuclei were stained with DAPI (b, d, f) or were not stained with DAPI for clarity (a, c, e). Images are of 60x magnification.

Next, we investigated the effect of C12-HSL on cytoskeletal associated proteins in PCa and normal epithelial cells (Figure 6(b)). Cofilin1 protein expression was observed in all PCa including RWPE1 cells and did not vary in the presence of C12-HSL. DU145 and RWPE1 WT cells had IQGAP1 protein expression, which diminished in the presence C12-HSL. Other cells had very low IQGAP1 expression. Total RhoC protein increased by 1.3-fold in PC3, 1.4-fold in LNCaP, and marginally in DU145 cells in the presence of C12-HSL. Similarly, increase in total vinculin protein expression was also noted in the PC3 and LNCaP cells treated with C12-HSL. However, vinculin protein status did not change in DU145 and RWPE1 cells in the presence of C12-HSL. Graphical representation for protein fold changes is depicted in Figure 6(c). As vinculin stabilizes actin structures, we tested how early during C12-HSL treatment could alter the actin cytoskeleton in PC3 cells. Fluorescence microscopy analysis for actin in WT PC3 cells indicated an organized actin structure (Figures 7(a) and 7(b)). However, in the presence of C12-HSL cellular actin appears to have punctuated morphology suggesting C12-HSL effect on actin appear early (within one hour) during the treatment (Figures 7(c)–7(f)).

## 4. Discussion

The QS signaling molecule C12-HSL naturally produced in* pseudomonas aeruginosa* bacteria at *μ*M concentrations has demonstrated growth inhibitory activity in different cancer cells [3–7] and its action is selective for tumorigenic human cells [15]. Previous studies indicated that C12-HSL downregulated thymidylate synthase [29] in H630 cells and increased the efficacy for Taxol treatment. Growth inhibition in PC3 cells was also reported in the presence of C12-HSL [30]. However, the effect of C12-HSL on tumorigenic cells from different organs as well as cells within the same tumor type such as PCa with different characteristics (SCNC versus adenocarcinoma) and the nonneoplastic cell lines can be highly variable. Moreover, the effects of C12-HSL on signal transduction molecules and proteins that affect cellular properties including cellular adhesion and migration in PCa cells are less known.

C12-HSL at *μ*M concentrations may reduce the viability of mammalian cells [15]. Our findings that minimal C12-HSL effect with nontumorigenic epithelial cells is in keeping with previous reports that epithelial cells from breast [15] and Hep-2 epithelial cells [31] were tolerant to the changes induced by C12-HSL. The susceptibility of PCa cells to C12-HSL induced apoptosis were interesting. For instance, the SCNC (PC3) cells underwent more apoptosis compared to DU145 and LNCaP adenocarcinoma cells. This could be due to the difference in the susceptibility of the apoptotic machinery in these cells or due to diminished presence of signaling factors which trigger these events. At present, it appears that triggering of caspases is the prime mechanism in the initiation of apoptosis by C12-HSL as observed in this study and the previously reported studies [15, 24, 29]. Recently, it has also been shown that C12-HSL induces apoptosis independent of antiapoptotic Bc1-2 proteins as one of the possible mechanisms in lung tumor cells [26] likely due to mitochondrial membrane permeabilization. However, it is unclear whether this is a universal mechanism across different cell types or specific to certain cell types.

Epithelial cancer cells adapt rapidly to various microenvironments and can shift between alternate pathways that regulate survival, proliferation, and differentiation [32], so appropriate cell culture models are necessary to test the efficacy of novel drug-like compounds. In the current study, the cell behavior in a 3D Matrigel culture upon exposure to C12-HSL indicated a change in cellular morphology and viability of PCa cells as observed by phase contrast microscopy and in the presence of a cell permeable Calcein dye. Normal cells displayed a spheroid shape which did not alter much in the presence of C12-HSL and retained most of the dye. Thus, using the 3D culture to study the C12-HSL effect for target identification and as a platform for drug discovery allows exploring the complexity in preclinical drug development as well as compound efficacies before proceeding to* in vivo* studies.

STAT3 participates in different signaling pathways that promotes the progression of castration resistant prostate cancer (CRPC) [13]. Previous studies with breast carcinoma cells [15] indicated that STAT3 could be a molecular target for C12-HSL. However, STAT3 is not uniformly present in all the PCa cells. For instance, WT PC3 cells are null for STAT3 [33] and as indicated in results are more susceptible for apoptosis in the presence of C12-HSL. Other PCa cells including normal cells had varying levels of both STAT3 gene and protein expression and the extent of apoptosis varied between these cells. In DU145 cells both total STAT3 and pSTAT3 decreased upon treatment with C12-HSL, which could be likely a facilitating factor for apoptosis in these cells. A similar decrease in STAT3 was noted in DU145 cells when treated with Ginkgetin [34], a biflavone from Ginkgo biloba leaves. Also, studies in human and canine osteosarcoma cells treated with a curcumin analog FLLL32 displayed similar downregulation of total and pSTAT3 [35], possibly due to ubiquitin mediated degradation. However, no constitutive or C12-HSL dependent changes in STAT3 were observed in LNCaP or RWPE-1 cells.

C12-HSL addition induced mRNA expression and phosphorylation of STAT1 in PC3 and LNCaP cells. However, the total protein expression was relatively low, and no pSTAT1 was observed in DU145 cells relative to other PCa cells. STAT1 is the principal mediator of types I and II interferon activation [36]. STAT1 can regulate the expression of proapoptotic and genes associated with antiproliferation molecules such as caspases and CDKN1A [37]. Also, it has been reported that interleukin receptor-6, which can activate STAT3 predominantly, in the absence of the latter activates the STAT1 efficiently [36]. Our studies indicate that C12-HSL induces more STAT1 phosphorylation (Tyr701) in PC3 cells (STAT3 negative) compared to LNCaP (STAT3 positive) cells. Interestingly, interleukin-6 (IL-6) has been shown to decrease tumor growth in LNCaP xenograft models [38]. Although we did not test directly the presence of IL-6 in PCa cells, C12-HSL likely can contribute to the induction of IL-6 in PCa cells as previous reports indicate that this compound can induce IL-6 in airway epithelial cells [3].

CDKN1A plays a role in transcriptional regulation and modulation of apoptosis [39]. Our studies indicated high CDKN1A gene expression in LNCaP relative to PC3, DU145 including RWPE1 cells. However, protein expression was very low in DU145 cells compared to other PCa and normal cells with a notable increase in the total protein, specifically in LNCaP cells upon exposure to C12-HSL. C12-HSL induced pCDKN1A (Thr-145) was also observed in the PCa cells except DU145 and RWPE1 cells. Phosphorylation at Thr-145 of CDKN1A is catalyzed by AKT/PKB, which is a regulator of cell proliferation and cell survival [39]. Inhibition of AKT had only modest effect on CDKN1A phosphorylation in endothelial cells [40] and a partial inhibition of AKT was reported earlier in breast carcinoma cells by C12-HSL [15]. CDKN1A phosphorylation induced by C12-HSL might influence the localization of this protein as pCDKN1A can relocate from nucleus to cytosol where it can interact with several proteins, which again may be specific for different cell types [39].

We further investigated the effect of C12-HSL on other cytoskeletal proteins such as vinculin, IQGAP, RhoC, and cofilin1 expression in the PCa and normal cells. C12-HSL has been shown to affect the migration in Caco-2 cells by targeting GTPase activating protein, IQGAP1 [22]. Our study showed IQGAP1 mRNA and protein expression in WT DU145 and RWPE1 cells and was decreased upon treatment with C12-HSL, although the extent of decrease was more prominent in DU145 cells. Interestingly, the expression of this protein in PC3 and LNCaP cells was low, indicating a primary role for this protein in the migration of these cells may be minimal or none. Further, our studies indicated no change in mRNA or protein expression of cofilin in PCa cells in the absence or presence of C12-HSL. Vinculin, an F-actin binding protein, has been suggested as a tumor suppressor by reducing tumor metastasis through decrease cell motility [41]. We observed that C12-HSL induces vinculin mRNA and total protein expression in PC3 and LNCaP cells but not in DU145 cells or RWPE1 cells. It has been shown previously that vinculin activating peptide sensitizes melanoma cells to dacarbazine chemotherapy [42]. Interestingly, another study reported that C12-HSL sensitizes Caco-2 cells to chemotherapy agents such as Taxol and 5-fluorouracil [13]. It is likely that C12-HSL could reduce cell motility in PCa cells by interfering with vinculin and other cytoskeletal proteins, although the effect could vary between the cells depending on the vinculin status (e.g., phosphorylation or autoinhibited) and their orientation with its protein partners. Like vinculin, we also observed an increase in RhoC mRNA and protein expression in all the cells tested except DU145 cells. RhoC has been implicated in control of cell motility, cell-cycle progression, adhesion, and apoptosis [43]. Contrary to the role of RhoC in tumor metastasis, its role in tumor growth is not univocal. For instance, induced RhoC expression did not have any effect on tumor growth in orthotopic lung cancer in mice [44]. We speculate that C12-HSL induction of RhoC in the PCa cells may play a primary role in cytoskeletal remodeling and along with other cytoskeletal proteins could affect cellular properties such as migration and adhesion. Along this line, to assess the effect of C12-HSL we tested cellular migration by wound healing assays and observed inhibition of cell migration in PCa cells as well as normal prostate cells. This property of C12-HSL should be taken into account if it were to be tested as an antitumorigenic agent* in vivo*. Thus the effect of C12-HSL on different cytoskeletal proteins such as IQGAP, RhoC, and vinculin could collectively contribute for the inhibition of cell motility as the mechanical link between these proteins may coordinate the overall effect of cell adhesion and migration properties.

In conclusion, our study demonstrates that C12-HSL affects the viability and colony formation of PCa cells by reducing STAT3 or increasing STAT1 activation depending on the cell type. Further, cellular migration could also be affected in the presence of C12-HSL through modulation of cytoskeletal proteins vinculin, RhoC, and IQGAP1. The viability of tumor cells grown in 3D matrix was also found to be affected by C12-HSL, which makes it a potential compound to be further explored for studying* in vivo* antitumorigenic properties in animal models.

## Figures and Tables

**Table 1:** EC_50_ values for viability, cytotoxicity, and apoptosis in normal and different PCa cells upon treatment with C12-HSL for 24 h. Values represent C12-HSL concentrations (*μ*mol/L).

Cells	Viability	Cytotoxicity	Apoptosis
RWPE1	>100	≥100	100
PC3	75	60	75
LNCaP	100	75	80
DU145	>100	80	82

## Supplementary Materials

Supplementary Materials Supplementary Figure 1 (Figure S1):* cell viability, cytotoxicity, and apoptosis upon C12-HSL treatment*. RWPE1, PC3, DU145, and LNCaP cells (5 × 10^3^ cells/well) were treated with C12-HSL at different concentrations ranging from 0 to 200 *μ*mol/L and incubated for 24 h. Viability, cytotoxicity, and apoptosis were measured using a fluorescence and luminescence assays.
